# Daytime Sleepiness Among Healthcare Workers Following SARS-CoV-2 Infection

**DOI:** 10.1192/j.eurpsy.2025.2325

**Published:** 2025-08-26

**Authors:** E. Bechrifa, D. Brahim, N. Mechergui, I. Youssef, M. Mersni, G. Bahri, H. Ben Said, M. Bani, N. Laadhari

**Affiliations:** 1 Occupational Medicine Department, Charles Nicolle Hospital; 2 Occupational Medicine Department, Habib Thameur Hospital; 3 Occupational Medicine Department, Mongi Slim Hospital; 4 Occupational Medicine Department, Maternity and Neonatology Center, Tunis, Tunisia

## Abstract

**Introduction:**

COVID-19 infections had a variety of symptoms, including a range of sleep disorders such as daytime sleepiness.

**Objectives:**

The objective of the study was to determine the frequency and persistence of daytime Sleepiness among HCWs post-COVID-19 and analysis of related risk factors.

**Methods:**

A prospective descriptive study was conducted over six months (January-July 2022) among HCWs at Charles-Nicolle Hospital in Tunis who contracted COVID-19. Daytime sleepiness was evaluated at three intervals: during the infection (T0), at three months, and at six months post-infection, using the Epworth Sleepiness Scale (ESS) in its French version. Anxiety and depressive symptoms were assessed with the Hospital Anxiety and Depression Scale (HAD) to examine their association with sleep disturbances.

**Results:**

The study included 155 HCWs, with an average age of 40.2 ± 10.3 years and an average work seniority of 14,1 ± 10 years. The assessment of anxiety-depressive disorders in the study population using the HAD scale showed that anxiety symptoms were certain in 27.3% of the personnel, while depressive symptoms were certain in 21.4% of cases. Additionally, 48.1% of participants showed no signs of anxiety, with 24.7% exhibiting doubtful symptoms. For depression, 59.1% had no symptoms, and 19.4% presented with doubtful symptomatology.

àAt baseline (T0), 47.1% of healthcare workers reported normal levels of sleepiness, 16.8% had moderate sleepiness, and 36.1% experienced abnormal sleepiness. By three months, these levels shifted, with 20.6% reporting normal sleepiness, 21.9% reporting moderate sleepiness, and 11.6% experiencing abnormal sleepiness. At six months, 7.7% reported normal sleepiness, 3.9% had moderate sleepiness, and 6.5% continued to experience abnormal sleepiness. Factors significantly associated with prolonged daytime sleepiness included obesity (p = 0.005), thrombophilia (p = 0.004), non-medical professional category (p = 0.019), hospitalization requiring oxygen therapy (p < 10⁻³), and the death of a close relative due to COVID-19 (p =0.016). Anxiety and/or depression, as assessed by the HAD scale, were also significantly associated with persistent sleepiness (p = 0.03).

**Conclusions:**

Persistent daytime sleepiness among HCWs post-COVID-19 infection highlights the need for targeted interventions focused on sleep quality and mental health support, which could enhance well-being and facilitate their occupational reintegration.

**Disclosure of Interest:**

None Declared

